# Biomaterials Development, Modification, and Potential Application for Interventional Cardiology

**DOI:** 10.1155/2020/4890483

**Published:** 2020-02-28

**Authors:** Jingan Li, Salvatore De Rosa, Juan Wang, Kun Zhang

**Affiliations:** ^1^Zhengzhou University, Zhengzhou, China; ^2^“Magna Graecia” University, Catanzaro, Italy; ^3^Yale University, New Haven, USA

Vascular stent interventional therapy is the main approach for the clinical treatment of coronary artery diseases. However, due to the insufficient biocompatibility of cardiovascular materials, the implantation of stents often leads to serious adverse cardiac events. Neointimal hyperplasia contributes to the pathophysiological process of several different vascular disorders, such as restenosis after angioplasty, allograft vasculopathy, vein graft stenosis, and atherosclerosis. Many novel biomaterials such as nanoparticles have attracted tremendous interest. In this special issue, we intend to cover recent experimental progress on the modification of stent materials. The purpose of this special issue is to present the recent progress in biomaterials development, modification, and potential application for interventional cardiology. A brief summary of all accepted papers is provided below.

The paper reported by X. Yue et al. evaluated the mechanical performances of the existing four types of DES with the length of 38 mm, including crossing ability, compliance, elastic recoil, and longitudinal strength. Here, the 38 mm long stents from XIENCE Xpedition (Abbott, US), SYNERGY (Boston Scientific, US), FIREHAWK (Microport, China), and HELIOS (HELIOS, China) were collected. The results indicated that the stents from XIENCE Xpeditionand SYNERGY performed the best crossing ability. The reduced ratio of stent diameter from XIENCE Xpedition was the least, indicating better compliance. In addition, the elastic recoil percentage of stents revealed better elastic recoil in the stent from SYNERGY. Moreover, the stent from XIENCE Xpedition had less displacement under pressure with the best longitudinal strength. The evaluation of mechanical properties for the stent with 38 mm including crossing ability, compliance, elastic recoil, and longitudinal strength for long stents could provide a reference index for the clinical application.

In the paper by S. Liu et al., they have reported the novel fibronectin- (Fn-) loaded poly-L lysine/heparin nanoparticles constructed for stent surface modification. In vitro blood compatibility and in vitro cellular compatibility evaluations were tested. It is found that the incorporation of Fn can improve the binding density of nanoparticles, which may contribute to enhancing the anticoagulant properties of the surface and thereby prevent the coagulation reaction caused by the presence of Fn. In addition, the Fn-loaded nanocoating was found to effectively improve the adhesion and proliferation of vascular endothelial cells on the material surface and thereby accelerate endothelium regeneration. The results showed that the nanoparticles-modified surface could effectively reduce platelet adhesion and activation and provide adequate efficacy in promoting the adhesion and proliferation of endothelial cells and thereby accelerate endothelialization. Based on the structure and function of the extracellular matrix on vascular injury healing, it provides a new approach for the surface biological function modification of vascular stents.

In the paper by Z. Huang et al., they have reported the capability of hydroxyapatite nanoparticles to deliver drugs. Composites containing drug delivery compounds were synthesized by coprecipitation and freeze-drying and then characterized by scanning electron microscopy, X-ray diffraction, and Fourier transform infrared spectroscopy. The use of hydroxyapatite nanoparticles (nano-SHAP) alone did not affect the proliferation of normal cell lines. However, nanoparticles containing the different amounts of norcantharidin in the composite materials had different inhibitory effects on osteosarcoma and different proliferative effects on osteoblasts. Also, with the increase of the norcantharidin dose, the antitumor performance of the composite has been enhanced. In summary, the nano-SHAP system can inhibit the growth of tumors and induce the proliferation of osteoblasts.

The work by Y. Wang et al. has modified biscarbamate cross-linked polyethylenimine derivative (PEI-Et) through PEGylation and thus obtained polyethylene glycol-graft-polyethylenimine derivative (PEG-Et 1:1), which has lower cytotoxicity and higher gene transfection efficiency compared with PEI-Et. In this study, PEG-Et 1:1 was employed in Smad3 shRNA (shSmad3) delivery for preventing intimal hyperplasia after vascular injury. It was observed that PEG-Et 1:1 could condense shSmad3 gene into nanoparticles with the particle size of 115–168 nm and zeta potential of 3–6 mV. PEG-Et 1:1 displayed remarkably lower cytotoxicity, higher transfection efficiency, and shRNA silencing efficiency than PEI-Et and PEI 25 kDa in vascular smooth muscle cells. Moreover, PEG-Et 1:1/shSmad3 polyplex treatment significantly inhibited collagen, matrix metalloproteinase 1 (MMP1), MMP2 and MMP9 expression, and upregulated tissue inhibitor of metalloproteinase 1 (TIMP1) expression both in vitro and in vivo. Furthermore, intravascular delivery of shSmad3 with PEG-Et 1:1 polyplex efficiently reduced Smad3 expression and inhibited intimal thickening 14 days after vascular injury. Ultimately, this study indicated that PEGEt 1:1-mediated local delivery of shSmad3 is a promising strategy for preventing intimal thickening.

The paper reported by S. Mosbahi et al. aimed to enhance the antiosteoporotic performance of bioactive glass (46S6) through its association with bisphosphonate such as risedronate. In vitro and in vivo explorations have been carried out. The association of bioactive glass and risedronate has been performed by the adsorption process. Structure analyses have been carried out to evaluate and to understand the chemical interactions by the Solid Nuclear Magnetic Resonance and the spectra deconvolution. In vitro experiments showed the enhancement of the chemical reactivity of the composites 46S6-xRIS compared with the pure bioactive glass. In vivo results showed good behavior with only 8% of introduced risedronate in the glass matrix.

